# Isolation and Identification of Chicken-Derived Lactic Acid Bacteria: In Vitro Probiotic Properties and Antagonistic Effects against *Salmonella pullorum*, *Staphylococcus aureus*, and *Escherichia coli*

**DOI:** 10.3390/microorganisms12040795

**Published:** 2024-04-15

**Authors:** Congcong Tian, Lei Wang, Mengjian Liu, Jiancheng Liu, Mingxin Qiu, Yong Chen

**Affiliations:** Xinjiang Key Laboratory of Herbivore Nutrition for Meat & Milk, College of Animal Science, Xinjiang Agricultural University, Urumqi 830052, China; tiancc2023@163.com (C.T.); xjauwl@163.com (L.W.); liumengjian@xjau.edu.cn (M.L.); liujc@xjau.edu.cn (J.L.); xjauqmx@163.com (M.Q.)

**Keywords:** probiotic, lactic acid bacteria, antibacterial activity, stress tolerance, *Lactiplantibacillus pentosus*, pathogens, chicken cecum microbiota

## Abstract

The development of probiotics capable of quickly colonizing the intestines of animals is important in promoting the healthy growth of livestock. The aim of this study was to screen lactic acid bacteria (LAB) from the intestinal microbiota of chickens with potential applications, and to evaluate their probiotic properties and antagonistic abilities against *Salmonella pullorum*, *Staphylococcus aureus*, and *Escherichia coli*. The results showed that a total of 79 strains with the characteristics of LAB were isolated from the chicken cecum microbiota, of which 7 strains exhibited strong inhibitory activity against *S*. *pullorum*, *S. aureus*, and *E. coli*. Performing 16s rDNA sequencing revealed that these seven strains were *Lactiplantibacillus pentosus* (*n* = 1), *Lactiplantibacillus plantarum* (*n* = 3), *Lactiplantibacillus paraplantarum* (*n* = 1), *Lactiplantibacillus argentoratensis* (*n* = 1), and *Lactiplantibacillus fabifermentans* (*n* = 1). Among them, *L. pentosus* R26 and *L. plantarum* R32 exhibited superior antibacterial activity. These two strains demonstrated high lactic acid production ability, with survival rates of 86.29% and 87.99% after 3 h of treatment at pH 1.5, 86.66% and 85.52% after 3 h of treatment with 0.5% bile salts, 90.03% and 88.16% after 2 h of treatment with simulated gastric fluid, and 98.92% and 98.22% after 2 h of treatment with simulated intestinal fluid, respectively. Co-cultivation with *L. pentosus* R26 for 24 h resulted in 50% of the pathogens being antagonized, while almost complete inhibition was observed following 72 h of co-cultivation. In conclusion, *L. pentosus* R26 and *L. plantarum* R32 exhibited high antibacterial activity and acid production capability, while also demonstrating satisfactory tolerance to low pH values and high concentrations of bile salts and digestive fluid. The probiotic characteristics and stress resistance of *L. pentosus* R26 were slightly superior to those of *L. plantarum* R32, indicating its potential for development as a probiotic.

## 1. Introduction

The gut microbiome is a complex ecosystem in which thousands of microorganisms live, both beneficial and potentially pathogenic, with beneficial bacteria playing an important role in the maintenance of host health [1]. The full performance potential of animals can only be achieved by protecting their health. Conversely, placing bans or strict restrictions on the use of antibiotics in animal feed to promote growth has led to strong interest in discovering alternatives. A growing number of studies have shown that the addition of probiotics to the diet strengthens immunity, maintains intestinal health, and increases animal performance by modulating the intestinal microbiota [2], improving the integrity of intestinal epithelial cells [3], increasing the production of immunoglobulin A (IgA) and antimicrobial peptides [4,5], as well as regulating the balance of bile acids in the body [6]. Therefore, probiotics are deemed to constitute an environmentally friendly and safe alternative to antibiotics.

The genus *Lactobacillus* is the predominant probiotic present in chicken intestines. It cannot be isolated from the cecum of newly hatched chickens. However, it has been found in the crop and cecum of 1-day-old chickens or 4 h after feeding, colonizing the duodenum, ileum, and cecum within 24 h after hatching [7]. At 3 d of age, *Lactobacillus* already accounts for 25% of the total bacteria in the content of the cecum. The relative abundance of *Lactobacillus* in the microbiota of the crop, gizzard, duodenum, jejunum, and ileum of adult hens exceeds 60% [8]. In addition to fermenting carbohydrates to produce lactic acid, *Lactobacillus* also generates bacteriocins or antimicrobial peptides, which exhibit favorable antibacterial effects against *Staphylococcus aureus*, *Escherichia coli*, *Salmonella typhi*, and other pathogenic bacteria [9]. The addition of *Lactobacillus rhamnosus* to the diet improves the cecal microbiota composition, and promotes intestinal development and epithelial maturation in broilers, thereby alleviating the intestinal dysfunction caused by hot stress [10]. Furthermore, adding *Lactobacillus acidophilus* to the diet increases body weight, feed intake, and average daily gain, upregulates occludin and ZO-1 gene expression, and increases the survival rate of broilers challenged with *E. coli* [11]. Likewise, dietary supplementation with *Lactobacillus plantarum* is more beneficial for the recovery of *Clostridium perfringens*-infected egg-laying chickens [12]. The addition of *Lactobacillus salivarius* to broiler diets not only improves growth performance, but also increases the number of beneficial bacteria present in the intestinal tract, such as *Lactobacillus* and *Bifidobacteria*, reduces the presence of harmful bacteria, such as *E. coli* and total aerobes, lowers the enzyme activity of harmful bacteria, and improves the morphology of intestinal tissues [13]. In addition, *Lactobacillus paracaseis sparacasei* and *L. rhamnosus* showed similar effects [14]. These facts suggest that *Lactobacillus* plays an important role in maintaining host intestinal health and improving livestock efficiency.

*Lactobacillus pentosus* is found in vegetables, fruits, fermented products, and the mammalian gut. Some *L. pentosus* strains possess health-promoting properties, such as immunomodulatory and antiproliferative activities, and are regarded as potential probiotic strains [15]. Several reports sustain that *L. pentosus*, both used alone or mixed with other probiotics in the diets of chickens and pigs, has the ability to improve growth performance, reduce diarrhea, increase survival rate, reduce the number of intestinal pathogens [16,17,18], and alleviate ulcerative colon inflammation in mice [19].

Little has been reported about *L. pentosus* of the animal intestinal origin. García-Hernández and coworkers isolated a strain of *L. pentosus* LB-31 from chicken feces that showed robust antimicrobial activity against *E. coli*, *Snterica serovar Typhimurium*, *Shigella flexneri*, *Shigella sonnei*, *Listeria monocytogenes*, and *Enterococcus faecalis* [20]. In addition to exhibiting impressive antimicrobial activity against pathogenic bacteria, *L. pentosus* isolated from chicken crop and cecal contents tolerated pH 3 and 0.3% bile salts [21]. Homologous probiotics display the best effectiveness of any bacterial preparations when acting on the host [22]. Therefore, the discovery of beneficial microorganisms from chicken intestines, with better probiotic properties and stress resistance, for use in poultry production will have robust and useful application prospects, being of great importance for ensuring animal health, improving production performance, and increasing the economic benefits of animal husbandry.

The objective of this study was to isolate and identify some novel *Lactobacillus* samples from broiler intestines with favorable acid production ability, antimicrobial activity, and stress tolerance in order to develop a novel probiotic for poultry production.

## 2. Materials and Methods

Experimental procedures which involved animals were approved by the Experimental Animal Welfare Ethics Committee of Xinjiang Agricultural University (protocol number: 20230405).

### 2.1. Pathogenic Bacteria, Medium, Simulated Gastric, and Small Intestinal Fluids

*E. coli* CVCC 1382, *S. aureus* CVCC 2257, and *Salmonella pullorum* CVCC 525 were purchased from the National Center for Veterinary Culture Collection. The de Man, Rogosa, and Sharpe (MRS) medium (6.8 ± 0.5), Salmonella Shigella (SS) agar medium (7.0 ± 0.2), eosin methylene blue (EMB) agar medium (pH 7.3 ± 0.5), nutrient agar (NA), and nutrient broth (NB) were prepared with reference to previous reports [23,24,25]. Simulated gastric fluid (10 g/L porcine pepsin, pH 1.5) and intestinal fluid (6.8 g/L KH_2_PO_4_, 10 g/L porcine pancreatin, pH 6.8) were prepared according to the *Pharmacopoeia of the People’s Republic of China* [26]. Reagents were purchased from Qingdao Hi-Tech Industrial Park Hope Bio-Technology Co., Ltd. (Qingdao, China), and Solarbio Science & Technology Co., Ltd. (Beijing, China).

### 2.2. Isolation of Lactic Acid Bacteria (LAB)

A healthy, 72-day-old yellow-feathered broiler was sacrificed via exsanguination from the jugular vein. The cecum was separated under aseptic conditions, and the contents in the cecum were carefully scraped out. In total, 1.0 g of cecum contents was added to 9 mL of 0.85% sterile saline and the product was stirred in a water bath shaker (SHZ-88, Jinyi Medical Technology Co., Ltd., Shanghai, China) at 170 r/min and 37 °C for 3 h. A volume of 1 mL of the mixture was inoculated into 100 mL of MRS broth, undergoing enrichment cultivation in an electric heating incubator (DHP-9162, Yiheng Scientific Instrument Co., Ltd., Shanghai, China) for 12 h at 37 °C. Later, 1 mL of the enrichment culture was sequentially diluted 10^4^ to 10^7^ times using sterile deionized water (dH_2_O). A double-layer agar method was utilized for the microaerophilic cultivation of LAB [27]. In brief, 100 μL of diluted culture was added to 10 mL of sterile MRS agar (base agar, at 40–50 °C), containing 0.75% CaCO_3_ as an indicator. The product was mixed thoroughly and immediately poured into a Petri dish. After the base agar had solidified, another 10 mL of MRS agar (top agar, at 40–50 °C) was added and used to cover the base agar completely. Once the top agar had solidified, the dish was incubated at 37 °C for 24 h. Colonies with the typical characteristics of lactic acid bacteria were picked [28] and successively propagated until we obtained single colonies. The purified single colonies were inoculated in 100 mL of MRS broth, incubated at 37 °C for 12 h, and then Gram stained [29]. The morphology of the strains was observed and recorded using an electron microscope with a magnification of 100× (ECLIPSE Ci-L, Nikon Corporation, Tokyo, Japan).

### 2.3. Antimicrobial Activity

The agar well diffusion method was used to evaluate the antimicrobial capacity of LAB [30]. Briefly, *E. coli*, *S. aureus* and *S. pullorum* were used as indicator bacteria. The pathogens were inoculated with NB in a water bath shaker (SHZ-88, Shanghai Jinyi Medical Technology Co., Ltd., Shanghai, China) at 37 °C and 170 r/min for 8 h until reaching the logarithmic growth phase. The concentration of the pathogens was adjusted to 1 × 10^8^ colony-forming units (CFU)/mL with NB [31]. After sterilization, the NA was cooled to approximately 40–50 °C, and the pathogens were inoculated at a concentration of 1% (*v*/*v*), thoroughly mixed, and then poured into Petri dishes. Then, 3 Oxford cups with diameters of 8 mm were evenly placed on the solidified NA at equal distances, and 200 μL of LAB inoculum was added to each cup. The dishes were allowed to equilibrate on a clean bench for 2 h, before being transferred to an incubator for overnight diffusion at 4 °C. This was subsequently followed by incubation at 37 °C for 10 h. Upon completion of the incubation, the diameters of the inhibition zones were measured using a digital vernier caliper with a cross-streaking method. The strain exhibiting the most prominent antimicrobial activity was chosen for use in subsequent experiments.

### 2.4. Molecular Identification

The top 7 strains with the highest antibacterial activity were selected and genomic DNA were extracted using an Ezup column-based bacterial genomic DNA extraction kit (Sangon Biotech Co., Ltd., Shanghai, China), with the procedure performed according to the manufacturer’s instructions. PCR of the 16S rDNA with primers 27F (5′-AGTTTGATCMTGGCTCAG-3′) and 1492R (5′-GGTTACCTTGTTACGACTT-3′) [32] used 0.5 μL of DNA template (20–50 ng/μL), 2.5 μL of 10 × PCR buffer (with 50 mol/L Mg^2+^), 1.0 μL of dNTP (2.5 mmol/L each), 0.2 μL of DreamTaq^TM^ DNA Polymerase (5 U/µL) (Thermo Scientific, Waltham, MA, USA), and 0.5 μL of primers (10 μmol/L each), and ddH_2_O was added to achieve a final volume of 25 μL. PCR was performed on a 2720 thermal cycler (Applied Biosystems, Foster City, CA, USA) based on the following procedure: 5 min of denaturation at 95 °C; 30 cycles of denaturation at 94 °C for 30 s; annealing at 57 °C for 30 s; extension at 72 °C for 90 s; and a final extension at 72 °C for 10 min. The amplified fragments were sequenced using a 3730XL sequencer (Applied Biosystems) by Sangon. The nucleotide sequences were aligned with the available sequences in the GenBank database through NCBI blasting (https://blast.ncbi.nlm.nih.gov/Blast.cgi, accessed on 19 June 2023), and a phylogenetic tree was constructed using MEGA software (version 11) [33] in order to determine the species of the bacterial strains.

### 2.5. Growth Curve and Lactic Acid Production

The most potent antibacterial strains were selected, namely R26 and R32. These were inoculated in 100 mL of MRS broth and static-cultured at 37 °C for 20 h. The optical density value of the inoculum was adjusted to 0.8 at 600 nm (OD_600_) (GeneQuant pro, GE Healthcare Life Sciences, Chicago, IL, USA) using MRS broth. At a 1% (*v*/*v*) ratio, the inoculum was inoculated into MRS broth and incubated at 37 °C for 48 h. Samples were collected at 0 and 1 h after incubating, which was followed by sampling 5 mL every 2 h to measure the OD_600_. The pH of the bacterial suspension was determined using a laboratory pH meter (FE20-Five Easy Plus, Mettler Toledo, Shanghai, China). A volume of 400 μL of bacterial suspension was centrifuged using a microcentrifuge (5415D, Eppendorf, Hamburg, Germany) at 15,000× *g* at 4 °C for 15 min, and 300 μL of the supernatant was collected to measure the lactic acid concentration using a lactate analyzer (LM5, Anolox Instruments, Stourbridge, UK).

### 2.6. Stress Tolerance

#### 2.6.1. Acid Tolerance

The acid tolerance test was conducted according to the method described by Sirisopapong et al. [34], albeit with some modifications. Briefly, the final pH of the MRS broth was adjusted to 1.5, 2.5, and 3.5 using 0.1 mol/L HCl, respectively. After sterilization, the inoculum (1 × 10^9^ CFU/mL) was inoculated at a concentration of 1% (*v*/*v*) and static-cultured at 37 °C for 3 h. After incubation, 1 mL of the culture was diluted with sterile dH_2_O from 10^4^ to 10^6^ times. Then, 100 μL of each dilution was spread on MRS agar and the product was incubated at 37 °C for 12 h. Then, single colonies were counted, and the survival rate was calculated. The number of bacteria is expressed as log CFU/mL.

$$\mathrm{Survival}\;\mathrm{rate}\;(\%)=\frac{{\log\;\mathrm{CFU}/\mathrm{mL}}_{\left(F\right)}}{{\log\;\mathrm{CFU}/\mathrm{mL}}_{\left(I\right)}}\times 100$$

where log CFU/mL_(F)_ means final bacteria number after the acid tolerance test and log CFU/mL_(I)_ means initial bacteria number before the acid tolerance test.

#### 2.6.2. Bile Salt Tolerance

The bile salt tolerance of the strains was conducted following the procedures described by Hu et al. [35]. Porcine bile salt was added to the MRS broth to achieve concentrations of 0%, 0.1%, 0.3%, and 0.5%. The inocula of the strains R26 and R32 (1 × 10^9^ CFU/mL) were inoculated in a 1% (*v*/*v*) ratio and static-cultured at 37 °C for 3 h. The survival rate of the strains was calculated using the method described above (Section 2.6.1).

#### 2.6.3. Tolerance to Simulated Gastric and Intestinal Fluid

The tolerance to simulated gastric and intestinal fluid of the strains was determined according to the method reported by Sağlam et al. [36]. Simulated digestive fluids were filtered through a membrane filter with a 0.22 μm pore size. The inoculum strains R26 and R32 (1 × 10^9^ CFU/mL) were inoculated into the simulated gastric and intestinal fluid at concentrations of 2% (*v*/*v*) and then static-incubated at 37 °C for 3 h. At hourly intervals, we took 1.0 mL of the samples and evaluated the survival rate using the method described above (Section 2.6.1).

### 2.7. Antagonistic Effect

To evaluate the antagonistic effect of strain R26 on pathogenic bacteria, the method described by Sika-Kadji et al. was employed [21]. The OD_600_ of inoculum of the strains R26, *E. coli*, and *S. pullorum* was diluted to 0.8 with the addition of MRS broth. For the control, 1.0 mL of the pathogens was inoculated into 9.0 mL of MRS broth. For the treatment, 1.0 mL each of R26 and the pathogens were inoculated into 8.0 mL of MRS broth. The samples were then statically cultured at 37 °C. Every 24 h, a 100 μL sample of the culture was collected and diluted from 10^4^ to 10^6^ times with dH_2_O. Then, 100 μL of each dilution was spread onto SS agar (for *S. pullorum*) or EMB agar (for *E. coli*). Following a 24 h incubation period at 37 °C, the viable counts of the pathogen were determined using the method described above (Section 2.6.1).

### 2.8. Statistical Analysis

One-way analysis of variance (ANOVA) was performed using the software IBM SPSS Statistics (version 22, SPSS Inc., Chicago, IL, USA), and the linear and quadratic effects of the predictor variable levels were determined by using a contrast of orthogonal polynomials. Multiple comparisons between the groups were performed using Duncan’s method. The significance level was set at *p* < 0.05.

## 3. Results

### 3.1. Isolation of LAB

**Figure 1:**
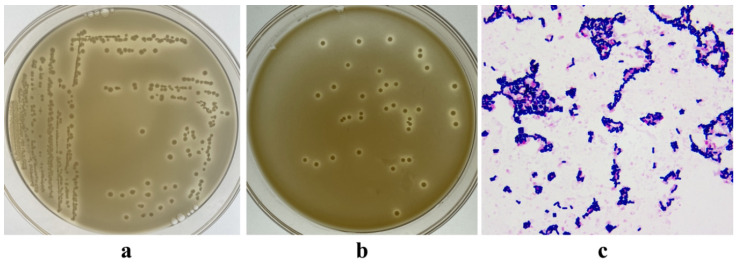
Morphological and Gram staining of isolated strains. (**a**,**b**): colony morphology of lactic acid bacteria in MRS agar (with 0.75% CaCO_3_). The colonies are milky white or light yellow, with a smooth surface and a slight elevation in the center. There is a distinct transparent halo of calcium solubilization around the periphery of the colony. (**c**): Gram staining of lactic acid bacteria. Cells are purple, rod-shaped, and without spores.

The morphological characteristics and Gram staining results of some strains cultured on MRS agar are shown in Figure 1a,b. Based on the typical morphological characteristics of LAB, a total of 79 strains were isolated. The candidate strains appeared purple after Gram staining and exhibited rod-shaped cells without spores when observed under a microscope (Figure 1c). The isolated bacteria were potentially identified as *Lactobacillus*.

### 3.2. Antibacterial Activity

**Figure 2:**
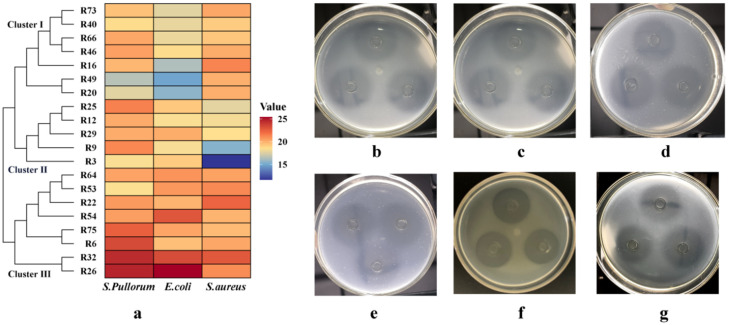
The inhibitory effect of probiotics on pathogenic bacteria. (**a**): cluster heatmap based on the inhibition zone of the top 20 strains with the largest antimicrobial activity against *S. pullorum*, *E. coli*, and *S. aureus*. (**b**–**d**): inhibition zones of the strain R26 against *S. pullorum*, *E. coli*, and *S. aureus*, respectively. (**e**–**g**): inhibition zones of the strain R32 against *S. pullorum*, *E. coli* and *S. aureus*, respectively.

The top 20 strains with the largest antimicrobial inhibition zones are displayed in Table 1. The strain R26 exhibited the largest diameter of inhibition zone against *S. pullorum* and *E. coli*, and this was significantly larger than that of the other strains (*p* < 0.05). The strain R32 also showed a significantly larger inhibition zone against the two pathogens compared to the remaining strains (*p* < 0.05). Regarding *S. aureus*, the strains R22 and R32 displayed the strongest antimicrobial activity, with their inhibition zone diameters being significantly larger than those of the other strains (*p* < 0.05). These were followed in strength by R16, R26, and R53, which also exhibited favorable antimicrobial activity against *S. aureus*. Cluster analysis based on the inhibition zones of these 20 strains revealed three clusters (Figure 2a), with Cluster III showing strong antimicrobial capabilities against all three pathogens. Among these eight strains, R26 (Figure 2b–d) and R32 (Figure 2e–g) exhibited the most prominent antimicrobial activity.

### 3.3. Molecular Identification

**Figure 3:**
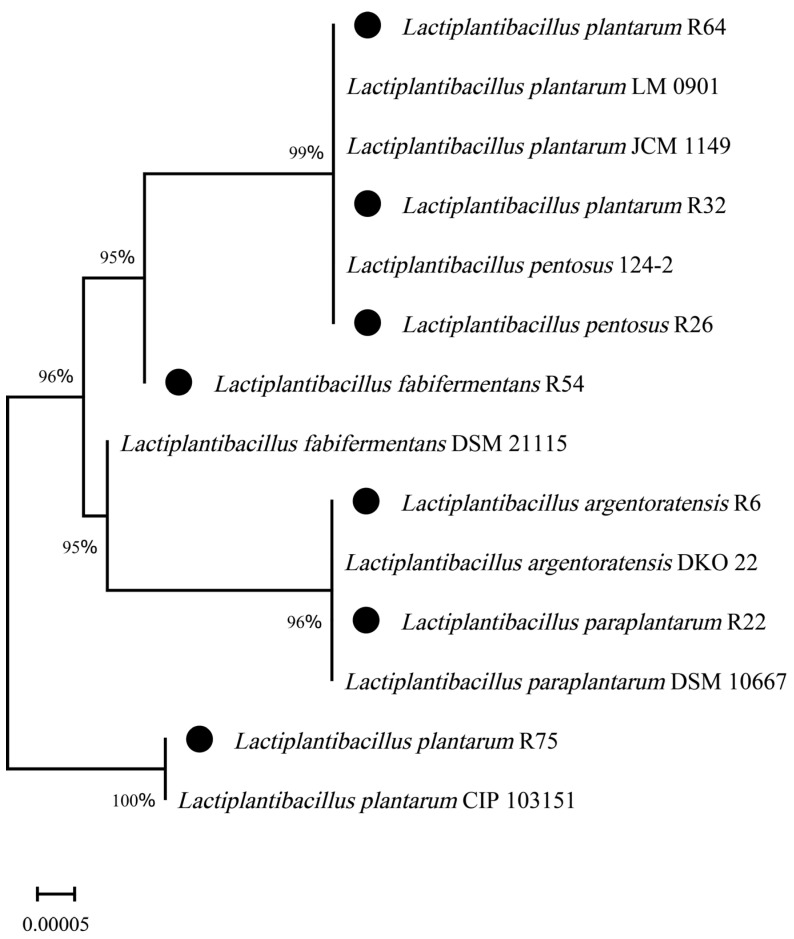
Unrooted polygenetic tree showing the relative positions of the seven strains isolated from the chicken cecum and their related species. The tree was constructed using the neighbor-joining method based on approximately 1500 bp of 16S rDNA sequences. Bootstrap values are shown at the branching points. Scale bars indicate genetic distance. (●) means strains identified in the present study.

Through 16S rDNA sequencing and DNA sequence alignment analysis, we found that the seven strains with strong antibacterial activity exhibited levels of identity >99% with *Lactiplantibacillus* (Table 2). These seven lactic acid bacteria included one strain of *Lactiplantibacillus pentosus*, three strains of *Lactiplantibacillus plantarum*, one strain of *Lactiplantibacillus paraplantarum*, one strain of *Lactiplantibacillus argentoratensis*, and one strain of *Lactiplantibacillus fabifermentans*. All sequences were submitted to GenBank and assigned accession numbers. The strain R26 was identified as *Lactiplantibacillus pentosus* (accession No. PP389393.1), and R32 was determined to be *Lactiplantibacillus plantarum* (PP390060.1). A phylogenetic tree was constructed based on the neighbor-joining method using MEGA 11 software, shown in Figure 3.

### 3.4. Growth Curve and Lactic Acid Production of L. pentosus R26 and L. plantarum R32

**Figure 4:**
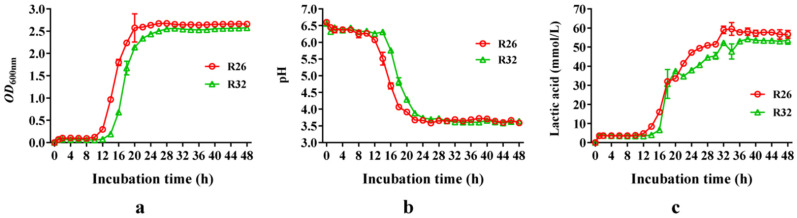
Growth curve, pH, and lactic acid concentration changes of *L. pentosus* R26 and *L. plantarum* R32. (**a**): growth curve (OD_600_); (**b**): pH of the medium; (**c**): concentration of lactic acid in the medium.

The lag phases of *L. pentosus* R26 and *L. plantarum* R32 were 10 h and 12 h, respectively, while *L. plantarum* R26 demonstrated a slightly faster growth rate (Figure 4a). With the extension of incubation time, lactic acid accumulated continuously in the medium, and the pH progressively decreased. *L. pentosus* R26 entered the stationary phase at 20 h of cultivation, with OD_600nm_ of around 2.6 and a pH of around 3.6. *L. plantarum* R32 also entered the stationary phase at 26 h, with OD_600nm_ of around 2.45 and a pH almost the same as that of *L. pentosus* R26 (Figure 4b). After 34 h of incubation, *L. pentosus* R26 exhibited the highest lactic acid concentration in the supernatant at 63.14 mmol/L, whereas *L. plantarum* R32 achieved this peak after 44 h of incubation, measuring 54.72 mmol/L (Figure 4c). This indicates that *L. pentosus* R26 has a superior growth rate and acid-producing ability compared to *L. plantarum* R32.

### 3.5. Acid Tolerance

As the pH of the culture medium decreased, the viable cell counts and survival rates of *L. pentosus* R26 and *L. plantarum* R32 fell significantly (*p* < 0.001) (Table 3). Under incubation at pH 3.5 for 3 h, the colony count for *L. pentosus* R26 was 7.95 log CFU/mL with a survival rate of 94.72%, while *L. plantarum* R32 had a colony count of 7.75 log CFU/mL with a survival rate of 92.23%. When the pH of the medium decreased to 1.5 after 3 h of incubation, the colony counts for *L. pentosus* R26 and *L. plantarum* R32 were 7.24 log CFU/mL and 7.40 log CFU/mL, with survival rates dropping to 86.29% and 87.99%, respectively. The pH value of the medium exhibited both linear and quadratic effects on the colony count and survival rate of *L. pentosus* R26 (*p* < 0.001), while it only showed a linear effect on *L. plantarum* R32 (*p* < 0.001). This indicates that both *L. pentosus* R26 and *L. plantarum* R32 exhibit favorable tolerance to low pH values.

### 3.6. Bile Salt Tolerance

Compared to the blank control, *L. pentosus* R26 showed no significant changes in colony count or survival rate after 3 h of cultivation in a medium containing 0.1% bile salts (*p* > 0.05). However, when the concentration of bile salt was increased to 0.3%, both the colony count and survival rate significantly decreased (*p* < 0.05) (Table 4). At a bile salt concentration of 0.5%, the colony count and survival rate decreased to 7.25 log CFU/mL and 86.66% (*p* < 0.05). *L. plantarum* R32 exhibited slightly lower bile salt tolerance than *L. pentosus* R26. At a bile salt concentration of 0.1% in the medium, both the colony count and survival rate significantly decreased (*p* < 0.05). When the bile salt concentration was reduced to 0.5%, the colony count and survival rate decreased to 7.13 log CFU/mL and 85.52% (*p* < 0.05). Bile salt exhibited both linear and quadratic effects on the colony count and survival rate of both the strains (*p* < 0.001).

### 3.7. Tolerance to Simulated Gastric and Intestinal Fluid

With the extension of cultivation time, both the colony count and survival rate of *L. pentosus* R26 and *L. plantarum* R32 exhibited decreases of varying degrees (Table 5 and Table 6). In terms of colony counts and survival rates, the strains showed better tolerance to simulated intestinal fluid than to simulated gastric fluid. In simulated gastric fluid, the incubation time exhibited linear and quadratic effects on both colony count and survival rate (*p* < 0.001), whereas in simulated intestinal fluid, it showed a linear effect (*p* < 0.001). After 1 h of incubation in simulated gastric fluid and intestinal fluid, the colony count and survival rate of *L. pentosus* R26 significantly decreased (*p* < 0.05). Similarly, after 0.5 h of incubation in simulated gastric fluid and 1 h in simulated intestinal fluid, the colony count and survival rate of *L. plantarum* R32 also fell significantly (*p* < 0.05). The survival rates of *L. pentosus* R26 after 2 h of treatment in simulated gastric fluid and intestinal fluid were 90.03% and 98.92%, whereas the survival rates of *L. plantarum* R32 were 88.16% and 98.22%, respectively.

### 3.8. Antagonistic Effect

**Figure 5:**
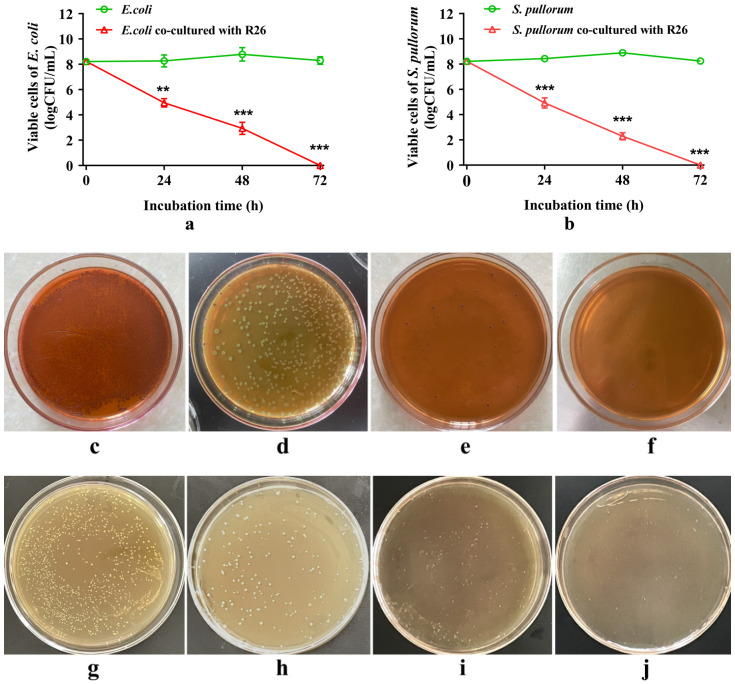
Co-cultured pathogenic bacteria with *L. pentosus* R26 in vitro. The pathogen was grown alone or co-cultured with *L. pentosus* R26 in MRS broth (1:1, *v*/*v*, OD600 was 0.8 each) for 0, 24, 48, and 72 h. After the appropriate dilution, the inoculum was cultured on eosin methylene blue (EMB) agar (for *E. coli*) or Salmonella Shigella (SS) agar (for *S. pullorum*) for 24 h. (**a**): changes in the viable cell count of *E. coli* after individual or co-culturing with *L. pentosus* R26, where the *X*-axis represents the incubation time (h), and the *Y*-axis represents the viable cell count of *E. coli* (lg(CFU/mL)). (**b**): changes in the viable cell count of *S. pullorum* when grown alone or co-cultured with *L. pentosus* R26, where the *X*-axis represents the incubation time (h), and the *Y*-axis represents the viable cell count of *S. pullorum* (lg(CFU/mL)). **: *p* < 0.01, ***: *p* < 0.001. (**c**–**f**): growth of *E. coli* on eosin-methylene blue (EMB) agar after co-culturing with *L. pentosus* R26 for 0, 24, 48, and 72 h, respectively. (**g**–**j**): growth of *S. pullorum* on Salmonella Shigella (SS) agar after co-culturing with *L. pentosus* R26 for 0, 24, 48, and 72 h, respectively.

When co-cultured with *L. pentosus* R26, both *E. coli* and *S. pullorum* exhibited a linear decline in viable cell counts (*p* < 0.001, linear effect). Compared to the strains grown alone, the viable cell counts of the pathogens co-cultured with *L. pentosus* R26 decreased significantly (*p* < 0.01). After 24 h co-culturing, the viable cell counts of *E. coli* and *S. pullorum* in the co-culture system were reduced by nearly 50% due to the presence of *L. pentosus* R26 (Figure 5a,b,d,h) (*p* < 0.01 and *p* < 0.001). By 48 h of co-culture, the viable cell counts of *E. coli* and *S. pullorum* decreased to around 100 CFU/mL (Figure 5a,b,e,i) (*p* < 0.001). By 72 h, the pathogens in the co-culture system were nearly completely inhibited (Figure 5a,b,f,j) (*p* < 0.001).

## 4. Discussion

Until March 2020, the genus *Lactobacillus* was viewed as encompassing a total of 261 species. These species exhibit extensive diversity at the phenotype, ecology, and genotype levels. Based on the comprehensive genome sequencing of the *Lactobacillus* genus, a recent reclassification effort partitioned the once-unified genus into a total of 25 separate genera [37]. Notably, *L. pentosus* and *L. plantarum* were found to belong to the same group as *Lactiplantibacillus*. Due to their promising probiotic properties and the safety of their use, *Lactiplantibacillus* found extensive application within various fields of human and animal healthcare, making it one of the most attractive alternatives to antibiotics.

In the animal gastrointestinal tract, naturally occurring probiotic bacteria play a crucial role in maintaining the stability of the gut microbiota and ensuring the health of the host. Typically, the contents of the small intestine, cecum, and feces serve as primary sources [38]. The cecum harbors a more diverse, rich, and stable microbiota compared to the ileum [39]. Therefore, this study opted to screen probiotic bacteria from the contents of the cecum in the hopes of identifying novel probiotic strains.

Antimicrobial activity is an important characteristic in the selection of probiotics. Produced by LAB, metabolites such as bacteriocins, organic acids, and exopolysaccharides can inhibit pathogens in the gut, preventing the colonization of pathogens in the intestines, maintaining the stability of the intestinal microbiota, and preventing and alleviating diseases caused by pathogens, such as diarrhea. Additionally, they help to maintain the intestinal epithelial barrier and regulate the host immune response [40,41]. *Lactobacillus* exhibits antimicrobial effects against various pathogens such as *S. citreus*, *E. coli*, *M. luteus*, and *Salmonella* [42,43]. In this study, several *Lactiplantibacillus* strains isolated from the intestines of healthy broiler chickens displayed strong inhibitory activity against *E. coli*, *S. pullorum*, and *S. aureus*. This is closely related to the bacteriocins produced by *Lactobacillus*. Bacteriocins are proteins or peptides with broad-spectrum antimicrobial activities. Some bacteriocins exhibit favorable thermal stability. The primary mechanisms of action against pathogens involves disrupting cell membranes, interfering with septum formation, blocking replication and transcription processes, suppressing protein synthesis, and inhibiting the synthesis of cell wall components (peptidoglycan units) [44].

After entering the host’s gut, probiotics usually need to quickly colonize and reproduce in order to gain an advantage in the microbial competition and become a dominant microorganism [45]. In this study, *L. pentosus* R26 showed rapid growth by entering the logarithmic phase after 10 h and reaching the platform phase after 18 h, demonstrating its superior growth rate compared to *L. plantarum* R32. Furthermore, *L. pentosus* R26 exhibited greater acid-producing capabilities than *L. plantarum* R32, which aided in preserving an enabling environment and inhibiting the growth of harmful bacteria.

Probiotics are required to establish colonization within the intestines, relying on their ability to endure and survive in the demanding intestinal environment to do so. In particular, they must exhibit resistance to the erosive effects induced by potent acidic gastric fluids. Consequently, only probiotics possessing strong resistance and exceptional survival rates can effectively perform their functions in the gut. *L. pentosus* ZFM94, isolated from the feces of healthy infants, demonstrated survival rates of 30.59% and 23.52% after exposure to simulated gastrointestinal juices with a pH value of 2.0 for 2 h and 4 h, respectively [43]. Several strains of LAB, which had been isolated from fermented foods and feeds, exhibited a survival rate ranging from 70% to 82% after 2 h treatment at a pH of 2.5 [46]. In this experiment, *L. pentosus* R26 showcased a high survival rate of 86.19% following a 3 h incubation period at pH 1.5. This suggests that *L. pentosus* R26 possesses exceptional acid resistance capabilities. LAB employ a vibrant array of acid resistance mechanisms. These include generating alkaline substances via the arginine dihydrolase system to neutralize acidic compounds, creating biofilms to protect cells from adverse environments, and effectively regulating intracellular and extracellular H^+^ concentrations through proton pumps [47].

Different parts of the animal gastrointestinal tract contain certain amounts of bile salts. These typically range in concentration from 0.03% to 0.3% [48]. Bile salts are substances with detergent-like activity that are formed by the combination of bile acids secreted from liver cells with either glycine or taurine to form sodium or potassium salts. Bile salts can disrupt the structure of microbial cell membranes and cause DNA damage, indicating strong antimicrobial activity [49]. Therefore, in order for probiotics to enter the hindgut and exert their effects, they must exhibit tolerance to bile salts. The survival rates of *L. pentosus* ZFM94 after 2 h of treatment with 0.1%, 0.2%, and 0.3% bile salts are 89.20%, 74.00%, and 63.20%, respectively [42]. In this study, the survival rates of *L. pentosus* R26 and *L. plantarum* R32 in 0.5% bile salt for 3 h were both over 85%, indicating that these two strains have strong bile salt tolerance. *Lactobacillus* and *Bifidobacteria* have been found to produce bile salt hydrolases and bile salt transporters, counteracting the negative effects of bile salts, and resisting the effects of bile salts on cells by enhancing their ability to maintain intracellular homeostasis and altering the structure and composition of their cell membranes [49,50]. Abedi and his colleagues analyzed the genome of *L. pentosus* CF2-10N and found that its fermentation enzymes could degrade complex substrates and contain genes for high bile salt tolerance [51]. Furthermore, the findings from this study demonstrated that both *L. pentosus* R26 and *L. plantarum* R32 exhibited high survival rates, exceeding 88%, following 2 h of exposure to simulated gastric fluid. Additionally, their survival rates remained above 98% after 2 h of exposure to simulated intestinal fluid, indicating their promising ability to accommodate the complex gastrointestinal environments.

Co-culturing probiotics and pathogens is an effective method to evaluate the inhibitory ability of the former against the latter. Ben and his colleagues found that *L. rhamnosus* LBF 16 and *L. paracasei* LBF 19 exhibited the complete inhibition of *E. coli* when co-cultured [52]. Wang et al. [53] reported that *L. plantarum* completely inhibited the growth of enterotoxigenic *E. coli* after 24 h of co-culturing. In this study, it was observed that when *L. pentosus* R26 was co-inoculated with *E. coli* in a ratio of 1:1, significant inhibition against the pathogenic bacteria was achieved after 24 h of co-culturing. *E. coli* was almost eliminated after 72 h. This suggests that *L. pentosus* R26 exhibits a beneficial antagonistic effect against pathogenic bacteria in vitro and possesses the potential to replace antibiotics. The production of lactic acid and antibacterial substances serves as the primary mechanism of antibacterial action for lactic acid bacteria. Additionally, metabolic crowding and the uptake of growth-supporting factors from nearby *E. coli* cells are also mechanisms for inhibiting pathogen growth [54,55].

*Lactobacilli* isolated in the present study demonstrated satisfactory stress resistance and antimicrobial ability in vitro. Further research is needed to determine if they cause a similar performance in vivo. Research has found that a *Limosilactobacillus reuteri* strain isolated from a mixture of feces, intestinal contents, and intestinal mucosa of chicken exhibited outstanding antibacterial activity against *S. pullorum* and fine tolerance to acid and simulated intestinal fluid. Animal experiments have shown that dietary supplementation with this strain improved weight loss, reduced the loads of *S. pullorum* in the intestine and viscera, and decreased intestinal inflammation of the broilers challenged with *S. pullorum* [56]. In the future, we will adopt animal experiments to test the effects of the *Lactobacilli* on the growth performance and intestinal health of chickens.

## 5. Conclusions

From a total of 79 isolated strains from a broiler cecum, seven strains of *Lactiplantibacillus* showing favorable antibacterial properties were isolated and identified from the cecal contents of chickens. Among them, *L. pentosus* R26 and *L. plantarum* R32 exhibited potent antibacterial activity against three pathogenic bacteria, an acid production capability, and demonstrated satisfactory tolerance to low pH, high concentrations of bile salts, and digestive juices. The probiotic characteristics and stress resistance of *L. pentosus* R26 were slightly superior to those of *L. plantarum* R32, indicating its potential for development as a probiotic.

## Figures and Tables

**Table 1:** Inhibition zones (mm) of chicken-derived LAB against pathogens.

Strains	*S. pullorum*	*E. coli*	*S. aureus*
R6	23.37 ± 0.15 ^c^	19.58 ± 0.07 ^f^	20.33 ± 0.15 ^cd^
R3	18.41 ± 0.0 ^l^	19.22 ± 0.03 ^g^	11.64 ± 0.05 ^j^
R9	21.24 ± 0.08 ^e^	18.32 ± 0.25 ^i^	15.43 ± 0.08 ^i^
R12	20.21 ± 0.02 ^h^	18.41 ± 0.16 ^ih^	18.25 ± 0.31 ^gh^
R16	19.83 ± 0.10 ^i^	16.35 ± 0.05 ^k^	21.35 ± 0.05 ^b^
R20	17.67 ± 0.12 ^m^	15.57 ± 0.08 ^e^	20.08 ± 0.03 ^cd^
R22	20.70 ± 0.10 ^i^	19.92 ± 0.13 ^d^	22.32 ± 1.17 ^a^
R25	21.44 ± 0.10 ^e^	19.30 ± 0.05 ^gf^	17.73 ± 0.12 ^h^
R26	24.72 ± 0.11 ^a^	25.45 ± 0.05 ^a^	21.11 ± 0.10 ^b^
R29	20.17 ± 0.12 ^h^	20.15 ± 0.05 ^e^	18.53 ± 0.06 ^g^
R32	24.48 ± 0.03 ^b^	23.32 ± 0.02 ^b^	22.80 ± 0.70 ^a^
R40	18.67 ± 0.06 ^k^	17.87 ± 0.06 ^j^	19.17 ± 0.15 ^f^
R46	20.50 ± 0.10 ^fg^	18.66 ± 0.12 ^h^	20.13 ± 0.15 ^cd^
R49	16.57 ± 0.12 ^n^	14.87 ± 0.31 ^m^	20.05 ± 0.13 ^cd^
R53	18.50 ± 0.10 ^lk^	20.77 ± 0.25 ^d^	21.21 ± 0.19 ^b^
R54	20.60 ± 0.10 ^f^	22.93 ± 0.32 ^c^	19.92 ± 0.13 ^cde^
R64	20.43 ± 0.05 ^fg^	20.89 ± 0.53 ^d^	20.45 ± 0.05 ^c^
R66	20.33 ± 0.29 ^gh^	17.97 ± 0.21 ^j^	19.37 ± 0.25 ^ef^
R73	19.52 ± 0.06 ^j^	17.67 ± 0.21 ^j^	20.31 ± 0.09 ^cd^
R75	22.53 ± 0.15 ^d^	20.38 ± 0.10 ^e^	19.70 ± 0.44 ^def^
*p* Value	<0.001	<0.001	<0.001

Note: Data are expressed as mean ± standard deviation (SD). The diameters of the inhibition zones do not include the diameters of the Oxford cups (Φ8.0 mm). In the same column, values with no letter or superscripts of the same letter indicate that there is no significant difference (*p* > 0.05), while those with different small-letter superscripts indicate significant differences (*p* < 0.05).

**Table 2:** Alignment analysis of isolated strains by 16S rDNA sequencing.

Strains (GenBank Accession No.)	The Highest Identity Strain (GenBank Accession No.)	Identity (%)
R6 (PP389395.1)	*Lactiplantibacillus argentoratensis* DKO 22 (NR 042254.1)	99.96
R22 (PP389397.1)	*Lactiplantibacillus paraplantarum* DSM 10667 (NR 025447.1)	99.80
R26 (PP389393.1)	*Lactiplantibacillus pentosus* 124-2 (NR 029133.1)	100.00
R32 (PP390060.1)	*Lactiplantibacillus plantarum* JCM 1149 (NR 117813.1)	99.00
R54 (PP390062.1)	*Lactiplantibacillus fabifermentans* DSM 21115 (NR113339.1)	99.23
R64 (PP390061.1)	*Lactiplantibacillus plantarum* LM 0901 (QQ569413.1)	99.93
R75 (PP389398.1)	*Lactiplantibacillus plantarum* CIP 103151 (NR 104573.1)	100.00

**Table 3:** Acid tolerance of *L. pentosus* R26 and *L. plantarum* R32.

Strains	Items	pH Value of the Medium				*p* Value		
		6.5	3.5	2.5	1.5	M	L	Q
*L. pentosus* R26	Colony count (log CFU/mL)	8.40 ± 0.03 ^a^	7.95 ± 0.02 ^b^	7.45 ± 0.04 ^c^	7.24 ± 0.02 ^d^	<0.001	<0.001	<0.001
	Survival rate (%)	100.00 ± 0.36 ^a^	94.72 ± 0.37 ^b^	88.69 ± 0.16 ^c^	86.29 ± 0.13 ^d^	<0.001	<0.001	<0.001
*L. plantarum* R32	Colony count (log CFU/mL)	8.41 ± 0.03 ^a^	7.75 ± 0.09 ^b^	7.51 ± 0.05 ^b^	7.40 ± 0.03 ^c^	<0.001	<0.001	0.140
	Survival rate (%)	100.00 ± 0.36 ^a^	92.23 ± 1.21 ^b^	89.33 ± 0.50 ^c^	87.99 ± 0.33 ^d^	<0.001	<0.001	0.151

Note: Data are expressed as mean ± standard deviation (SD). In the same row, values with no letter or the same letter superscripts mean no significant difference (*p* > 0.05), while those with different small-letter superscripts indicate significant difference (*p* < 0.05). M: main effects of pH value, L: linear of pH value, Q: quadratic effect of pH value.

**Table 4:** Bile salt tolerance of *L. pentosus* R26 and *L. plantarum* R32.

Strains	Items	Concentrations of Bile Salt (%)				*p* Value		
		0	0.10	0.30	0.50	M	L	Q
*L. pentosus* R26	Colony count (log CFU/mL)	8.37 ± 0.02 ^a^	8.33 ± 0.03 ^ab^	8.29 ± 0.02 ^b^	7.25 ± 0.03 ^c^	<0.001	<0.001	<0.001
	Survival rate (%)	100.00 ± 0.25 ^a^	99.56 ± 0.38 ^ab^	99.12 ± 0.30 ^b^	86.66 ± 0.52 ^c^	<0.001	<0.001	<0.001
*L. plantarum* R32	Colony count (log CFU/mL)	8.33 ± 0.01 ^a^	8.24 ± 0.03 ^b^	8.22 ± 0.01 ^b^	7.13 ± 0.02 ^c^	<0.001	<0.001	<0.001
	Survival rate (%)	100.00 ± 0.07 ^a^	98.92 ± 0.41 ^b^	98.60 ± 0.14 ^b^	85.52 ± 0.24 ^c^	<0.001	<0.001	<0.001

Note: Data are expressed as mean ± standard deviation (SD). In the same row, values with no letter or the same letter superscripts mean no significant difference (*p* > 0.05), while those with different small-letter superscripts indicate significant difference (*p* < 0.05). M: main effects of bile salt, L: linear of bile salt, Q: quadratic effect of bile salt.

**Table 5:** Tolerance to simulated gastric fluid of *L. pentosus* R26 and *L. plantarum* R32.

Strains	Items	Incubation Time (h)				*p* Value		
		0	0.5	1	2	M	L	Q
*L. pentosus* R26	Colony count (log CFU/mL)	8.36 ± 0.03 ^a^	8.32 ± 0.05 ^a^	8.22 ± 0.05 ^b^	7.53 ± 0.01 ^c^	<0.001	<0.001	<0.001
	Survival rate (%)	100.00 ± 0.43 ^a^	99.48 ± 1.01 ^ab^	98.29 ± 0.89 ^b^	90.03 ± 0.33 ^c^	<0.001	<0.001	<0.001
*L. Plantarum* R32	Colony count (log CFU/mL)	8.39 ± 0.04 ^a^	8.24 ± 0.01 ^b^	8.13 ± 0.03 ^c^	7.39 ± 0.03 ^d^	<0.001	<0.001	<0.001
	Survival rate (%)	100.00 ± 0.43 ^a^	98.17 ± 0.29 ^b^	96.86 ± 0.80 ^c^	88.16 ± 0.42 ^d^	<0.001	<0.001	<0.001

Note: Data are expressed as mean ± standard deviation (SD). In the same row, values with no letter or the same letter superscripts mean no significant difference (*p* > 0.05), while those with different small-letter superscripts indicate significant difference (*p* < 0.05). M: main effects of incubation time, L: linear of incubation time, Q: quadratic effect of incubation time.

**Table 6:** Tolerance to simulated intestinal fluid of *L. pentosus* R26 and *L. plantarum* R32.

Strains	Items	Incubation Time (h)				*p* Value		
		0	0.5	1	2	M	L	Q
*L. pentosus* R26	Colony count (log CFU/mL)	8.37 ± 0.01 ^a^	8.35 ± 0.01 ^a^	8.30 ± 0.02 ^b^	8.28 ± 0.01 ^b^	<0.001	<0.001	0.276
	Survival rate (%)	100.00 ± 0.12 ^a^	99.76 ± 0.07 ^a^	99.12 ± 0.36 ^b^	98.92 ± 0.24 ^b^	0.001	<0.001	0.407
*L. Plantarum* R32	Colony count (log CFU/mL)	8.42 ± 0.01 ^a^	8.38 ± 0.04 ^a^	8.31 ± 0.02 ^b^	8.27 ± 0.05 ^b^	0.002	<0.001	0.525
	Survival rate (%)	100.00 ± 0.12 ^a^	99.56 ± 0.49 ^a^	98.65 ± 0.27 ^b^	98.22 ± 0.74 ^b^	0.006	0.001	0.590

Note: Data are expressed as mean ± standard deviation (SD). In the same row, values with no letter or the same letter superscripts mean no significant difference (*p* > 0.05), while those with different small-letter superscripts indicate significant difference (*p* < 0.05). M: main effects of incubation time, L: linear of incubation time, Q: quadratic effect of incubation time.

## Data Availability

Data are contained within the article.
